# Human Experts and AI Models in Offender Risk Assessment: A Comparative Pilot Study Using the HCR‐20V3

**DOI:** 10.1002/bsl.70023

**Published:** 2025-11-11

**Authors:** Shai Farber

**Affiliations:** ^1^ Department of Law The Academic College of Law and Science Yezreel Valley Israel

**Keywords:** ChatGPT, Claude, Gemini, HCR‐20, human judgment, large language models, risk assessment

## Abstract

This pilot study compares offender risk assessments conducted by human experts and advanced large language models (LLMs) within the HCR‐20V3 framework. Both groups evaluated a series of synthetic forensic case vignettes designed to simulate realistic clinical conditions. Quantitative results indicate that AI models consistently assigned higher overall risk scores and demonstrated greater inter‐rater reliability compared to human assessors. Qualitative analysis revealed distinct reasoning patterns: AI systems emphasized historical and static risk factors and often recommended more intensive management strategies, whereas human experts focused on recent behavioral improvements, dynamic change, and rehabilitation potential. These contrasts highlight fundamental differences between algorithmic pattern recognition and human clinical judgment. The findings suggest that integrating AI‐generated analyses with professional expertise can enhance the consistency and transparency of risk evaluations, while preserving the ethical, contextual, and human‐centered insights essential to forensic and clinical decision‐making.

## 1 Introduction

### 1.1 Background on Risk Assessment in Clinical Criminology

Risk assessment in clinical criminology has been a cornerstone of criminal justice and mental health systems for decades, playing a crucial role in decision‐making processes such as sentencing, parole determinations, and treatment planning (Andrews and Bonta 2010). Over time, the field has evolved from relying solely on clinical judgment to incorporating structured professional judgment tools and actuarial methods (Douglas and Kropp 2002). The importance of accurate risk assessment cannot be overstated, as it directly impacts public safety, individual liberty, and the allocation of limited resources within these systems. However, traditional methods of risk assessment have faced criticism for potential biases, inconsistency, and limitations in predicting future behavior (Monahan and Skeem 2016).

In recent years, artificial intelligence (AI) has emerged as a powerful tool across various fields, including psychology and criminology. The application of AI in psychological evaluations marks a significant shift in how mental health professionals and criminologists approach assessment and decision‐making (Berk et al. 2018; Elyosef et al. 2024). AI systems, particularly those utilizing machine learning and natural language processing, have demonstrated the ability to analyze vast amounts of data, identify patterns, and make predictions. In the context of risk assessment, AI offers the potential to process and integrate complex information from diverse sources, which could lead to more accurate and consistent evaluations. However, integrating AI into psychological and criminological assessments also brings challenges. Researchers and practitioners have raised concerns about algorithmic bias, transparency, and the ethical implications of using AI in high‐stakes decision‐making processes (Chouldechova and Roth 2020; Elyosef et al. 2024).

This convergence of psychology, criminology, and artificial intelligence represents an emerging interdisciplinary frontier. It enables the integration of clinical reasoning, behavioral science, and computational analytics, thereby creating new opportunities to examine the interaction between human judgment and algorithmic processes in forensic contexts.

### 1.2 Research Objectives and Questions

This study introduces an innovative approach by directly comparing risk assessments performed by advanced artificial intelligence (AI) large language models (LLMs) with those conducted by human experts in the field of clinical criminology, using the HCR‐20V3 assessment tool. In this context, ‘using the HCR‐20V3’ refers only to the conceptual structured‐professional‐judgment framework described in the academic literature, and does not involve providing the AI models with the HCR‐20V3 manual or any part of its text. [Correction added on 29 December 2025, after first online publication: The preceding sentence has been added]. While prior research has explored AI across various domains of risk assessment, few empirical studies have directly compared advanced AI models with traditional human assessments within the HCR‐20V3 framework.

By integrating a well‐established, structured professional judgment tool with cutting‐edge natural language processing technologies, this research provides new insights into both the potential and the limitations of combining technological precision with human clinical expertise. Through this comparative design, the study aims to demonstrate how AI systems can complement, rather than replace, human evaluators, ultimately informing the development of more accurate, consistent, and ethically grounded risk assessment protocols.

The integration of AI into criminological risk assessment presents both opportunities and challenges that require systematic investigation. Accordingly, this study aims to evaluate the efficacy, consistency, and comprehensiveness of AI‐based risk assessments in comparison with those conducted by trained human professionals. It further explores the reasoning patterns, strengths, limitations, and potential biases of AI models, while addressing the ethical considerations inherent in their use within forensic and mental health contexts. By doing so, the study contributes to the ongoing discourse on the responsible and transparent implementation of AI in psychological and criminological practice.

To achieve these objectives, the following research questions guide the inquiry:

1. Comparative Performance: How do risk assessments conducted by AI models compare with those performed by human experts in terms of accuracy, consistency, and comprehensiveness?
2. Reasoning and Decision Processes: What are the key differences in the factors considered and the reasoning processes employed by AI models versus human experts when conducting risk assessments?
3. Ethical and Practical Implications: What are the primary ethical concerns and limitations associated with the use of AI in criminological risk assessment, and how might these challenges be effectively addressed?

The following sections present the theoretical foundations and prior research on risk assessment and AI applications, followed by a detailed description of the study design, sample construction, and data analysis methods. The results section reports both quantitative and qualitative findings, and the discussion integrates these outcomes within the broader ethical and theoretical context of clinical criminology. The paper concludes with implications for practice and directions for future research.

## 2 Literature Review

### 2.1 Traditional Methods of Risk Assessment in Criminology

Risk assessment in criminology has undergone significant evolution over the past century. Historically, clinicians have relied on unstructured judgment, informed by experience and intuition (Monahan 1981), a practice widely used yet criticized for its inconsistency and bias. In response to these limitations, actuarial methods emerged in the mid‐20th century, relying on models to predict risk based on static factors like age, gender, and criminal history (Bonta and Andrews 2007). While these models offered more reliability than unstructured judgment, they were sometimes limited in flexibility and failed to account for individual differences. Consequently, structured professional judgment (SPJ) tools such as the HCR‐20 became prominent in the late 20th century. SPJ tools aimed to integrate clinical expertise with validated risk factors, providing a structured framework while allowing professional discretion in the final risk assessment (Hart and Logan 2011).

### 2.2 The HCR‐20 Assessment Tool

The Historical‐Clinical‐Risk Management‐20 (HCR‐20) is among the most extensively used structured professional judgment (SPJ) instruments in mental health. It evaluates 20 empirically supported risk factors distributed across three domains: historical, clinical, and risk management (Douglas et al. 2013; Douglas and Belfrage 2014). The tool offers a structured yet adaptable framework, enabling assessors to combine standardized criteria with professional discretion. Its design facilitates the integration of both static and dynamic risk indicators, thereby enhancing its applicability across diverse populations and institutional settings (Singh et al. 2011; Challinor et al. 2021; Chen et al. 2023; Brookstein et al. 2021).

Recent work has further explored the potential to standardize HCR‐20 risk ratings into defined levels of violent recidivism risk, aiming to enhance clarity, consistency, and utility in risk communication and legal decision‐making (van Dooren et al. 2024; de Vogel et al. 2022). This evolution reflects a broader movement toward refining SPJ tools to support evidence‐based practices in criminal justice and psychiatry.

### 2.3 AI Applications in Psychological and Criminological Assessments

The field of artificial intelligence has recently opened new avenues for criminological and psychological risk assessment, primarily through advancements in natural language processing (NLP) and machine learning (Apene et al. 2024). AI models can analyze large datasets, detect patterns, and support predictive analysis, potentially enhancing accuracy in recidivism and other risk predictions (Berk and Hyatt 2015; Berk et al. 2018; Mandalapu et al. 2023). AI has been used to identify complex patterns in textual data, such as assessing tone, sentiment, and linguistic patterns within digital communications for risk profiling (Techopedia 2023). Beyond risk assessment, AI has also begun to transform broader areas of medicine and evidence analysis, offering new tools for pattern recognition, injury classification, and automated interpretation of clinical data (Piraianu et al. 2023). These advancements build on foundational work that demonstrates large language models, such as GPT‐3, are capable of performing complex linguistic tasks through few‐shot learning, enabling broad applicability without domain‐specific fine‐tuning (Brown et al. 2020).

A comprehensive systematic review by Dakalbab et al. (2022) examined recent developments in AI‐based crime prediction tools, identifying a wide range of applications—from recidivism forecasting to predictive policing—and highlighting both the technical promise and ethical limitations of these tools. This review highlights the growing role of AI in decision‐making and lays the groundwork for exploring more advanced language‐based models in this context. Similarly, Jenga et al. (2023) conducted an extensive review of machine learning applications in crime prediction, classifying algorithmic approaches used for various criminal offenses and evaluating their predictive accuracy. Their findings highlight the growing sophistication of AI tools in analyzing behavioral, geographic, and temporal data for risk estimation. These developments strengthen the theoretical and practical foundation for applying AI in forensic assessments.

Research led by Elyosef and colleagues has highlighted the growing potential of AI models in mental health and risk assessments. For example, Elyosef et al. (2024) examined the democratization of mental health services through AI, emphasizing how AI models could increase accessibility in underserved communities while addressing ethical challenges, such as bias and value alignment. Another study by Elyosef and Levkovich (2023) explores the application of ChatGPT in suicide risk assessment, demonstrating that while LLMs may align with clinical assessments in identifying high‐risk cues, they may lack sensitivity to complex human emotional states. Additionally, Hadar‐Shoval et al. (2024) assessed LLM alignment with human values in mental health applications using Schwartz's theory of basic values, revealing that AI models must be carefully calibrated to align ethically with human values for reliable clinical integration.

These studies highlight AI's ability to support psychology in tasks such as criminal profiling and mental health risk assessment. However, Elyosef et al.’s work also cautions that LLMs, while valuable as supportive tools, function best when paired with human expertise, particularly in cases where nuanced, context‐dependent interpretations are essential (Elyosef et al. 2024).

### 2.4 Ethical Considerations in AI‐Assisted Risk Assessment

The integration of AI into risk assessment practices raises significant ethical concerns. A primary issue is the potential for AI models to perpetuate historical biases embedded in training data, which could lead to discriminatory practices in criminology and mental health (Chouldechova 2017). Another challenge is the “black box” nature of many AI algorithms, which often lack transparency, making it difficult to interpret and verify the assessments they produce (Rudin 2019). Elyosef and colleagues advocate responsible AI frameworks in clinical settings, emphasizing transparency, accountability, and alignment with human values to mitigate ethical risks (Elyosef et al. 2024). Additionally, privacy concerns arise, as AI‐based assessments often require the collection and storage of sensitive personal data, raising issues of data security and individual privacy rights (Mittelstadt and Floridi 2016).

As AI technology continues to evolve, its application in criminological risk assessment must adhere to ethical principles, ensuring fairness, transparency, and respect for human rights. Elyosef's research further emphasizes the importance of balancing AI's consistency and analytical power with human judgment to maintain ethical integrity in high‐stakes applications (Elyosef et al. 2024).

## 3 Methodology

### 3.1 Research Design

The present research employed a mixed‐methods comparative design to evaluate differences between human and artificial intelligence (AI)‐based risk assessments in clinical criminology. The convergent parallel design (Creswell and Plano Clark 2018) allowed for the concurrent collection of quantitative and qualitative data, which were analyzed separately and then merged to provide a comprehensive understanding of the research problem. This mixed‐methods framework was particularly suited to the study's goals, as it enabled the integration of numerical consistency metrics with rich qualitative insights into reasoning patterns, thereby capturing both the structural and interpretive dimensions of risk assessment behavior. Given the highly sensitive nature of forensic case data and the strict privacy requirements in clinical criminology, the study employed a synthetic data generation methodology to ensure complete confidentiality while maintaining clinical relevance.

### 3.2 Sample Construction and Validation

The study was based on 10 meticulously crafted synthetic case reports (*N* = 10), yielding 60 risk assessments across six assessors (3 human evaluators and 3 AI models). The decision to employ synthetic data was guided by the highly sensitive nature of forensic case information and the ethical imperatives around privacy protection as per established professional guidelines (American Psychological Association 2017). This approach strikes a balance between the need for real‐world simulation and the ethical obligation to safeguard individual confidentiality, thereby preserving the validity of findings while avoiding the exposure of actual case details. Each synthetic case was constructed through a multi‐step process aimed at capturing realistic forensic complexity:

1. *Case Development*: We analyzed patterns within authentic clinical cases, focusing on common behaviors, risk profiles, and treatment histories. This informed the design of synthetic cases that integrate realistic combinations of risk and protective factors, closely mirroring the dynamics of actual scenarios.
2. *Clinical Validation*: Experienced clinical criminologists reviewed each case for relevance and fidelity to real‐world profiles. Feedback from this expert review informed further refinements, including adjustments to behavioral details and clinical descriptions, to enhance the case's authenticity.
3. *Ethical and Practical Standards*: The cases were structured around the HCR‐20V3, a widely accepted forensic risk management tool, and developed to align with ethical standards. This use of synthetic data not only maintains research integrity but also ensures compliance with privacy and confidentiality requirements in contexts.

The balanced selection of three AI models and three human evaluators enhances replicability and generalizability. This design enables robust comparative analysis and supports inter‐rater reliability assessments across both AI and human evaluators, thereby strengthening the study's capacity to explore consistency and variability in risk assessments.

*AI Models*: Three state‐of‐the‐art AI models were selected for the study, each representing the most advanced versions available as of September 2024: Claude AI (Anthropic), ChatGPT (OpenAI), and Gemini (Google). The choice of these specific AI models was driven by their advanced natural language processing (NLP) capabilities, demonstrated success in similar contexts, and their ability to process unstructured data effectively. Each model's distinct approach to language understanding allowed for a comparative analysis of varying AI methodologies, providing insights into the strengths and limitations of different technological frameworks.

*Data Collection Procedures*: The data collection yielded 60 comprehensive risk assessments, generated by six different assessors (three human experts and three AI models), evaluating 10 synthetic cases. Data collection proceeded in two parallel phases:

### 3.3 Human Assessments

Three clinical criminologists were selected as expert assessors according to stringent inclusion criteria. Each assessor had a minimum of eight years of experience in offender risk assessment, held current certification in the HCR‐20V3 instrument, and maintained active clinical practice within mental health settings. The group represented diverse areas of specialization, including violent offender management, rehabilitation programs, and substance abuse treatment. Each criminologist independently evaluated all 10 synthetic cases, resulting in a total of 30 human expert assessments (three assessors × 10 cases).

### 3.4 AI Assessments

The same 10 synthetic case reports were input into each of the three AI models, generating an additional 30 AI‐based assessments (3 AI models × 10 cases). To maintain consistency, the AI models were prompted with standardized instructions designed to replicate the role of a clinical criminologist. They did not have access to the human‐generated risk scores. This approach ensured that the AI assessments were based solely on the case information provided, allowing for an unbiased comparison with the evaluations of human experts.

### 3.5 Assessment Procedure

Both human assessors and AI models utilized the HCR‐20 Version 3 (HCR‐20V3; Douglas et al. 2013) as the framework for their evaluations. To avoid any misunderstanding, the AI models in this study were not trained using the HCR‐20V3 manual or any copyrighted content from it. Their assessments were based solely on the synthetic case vignettes and on general, non‐proprietary professional‐judgment instructions. [Correction added on 29 December 2025, after first online publication: The preceding sentence has been added]. The HCR‐20V3 is a comprehensive guide for violence risk assessment, encompassing 20 items across three domains: historical factors (10 items), clinical factors (5 items), and risk management factors (5 items). This tool was selected for its widespread use and validated reliability in forensic settings (Singh et al. 2011), ensuring a consistent basis for evaluating both AI and human assessments.

### 3.6 Data Analysis Methods

A structured content analysis was performed on all 60 assessments using a coding framework derived from the HCR‐20V3. The materials were examined by independent reviewers who were not informed whether each assessment had been produced by a human evaluator or an AI model, ensuring objectivity and minimizing potential bias. Inter‐rater agreement was subsequently reviewed to verify consistency across evaluations. In addition to the content analysis, basic statistical comparisons were conducted to examine differences in overall risk ratings between the two groups. To explore qualitative distinctions more deeply, a thematic analysis was carried out in accordance with Braun and Clarke's (2006) six‐phase approach, involving data familiarization, coding, theme development, and refinement.

### 3.7 Study Limitations

While designed to provide meaningful preliminary insights into AI‐assisted risk assessment, this pilot study acknowledges several methodological limitations. Although the total number of assessments was substantial (*N* = 60), these were derived from 10 synthetic cases, which limits the statistical generalizability of the findings. The use of synthetic data, while ethically necessary to ensure confidentiality, may not fully capture the interpersonal and contextual complexity characteristic of real forensic cases. Additionally, the number of human assessors was relatively small, which may not reflect the full range of clinical reasoning styles present in the broader professional community.

The AI models employed were general‐purpose systems not fine‐tuned for forensic or clinical contexts, which may have constrained their sensitivity to nuanced behavioral and contextual cues. Moreover, as large language models rely on probabilistic pattern recognition rather than genuine understanding, their reasoning processes may differ fundamentally from human cognitive judgment. Finally, given the rapid evolution of these models, with frequent version updates, future replications may yield differing results.

Taken together, these limitations underscore the exploratory and formative nature of the present research. They do not diminish its contribution but rather highlight the need for larger‐scale, domain‐adapted studies using real‐world forensic data to validate and extend these early findings.

## 4 Results

### 4.1 Quantitative Comparison of Risk Scores

The study employed a five‐point risk assessment scale, ranging from 1 (Low) to 5 (High), resulting in 60 comprehensive risk assessments derived from the evaluation of 10 synthetic cases. Each case received six independent evaluations: three from human clinical criminologists and three from AI models.

### 4.2 Overall Risk Score Comparison

Analysis revealed significant differences between the risk scores assigned by human assessors and those assigned by AI models (see Table 1).

**TABLE 1:** Comparison of mean risk scores.

Assessor type	Mean score	Standard deviation	95% CI
Human assessors	2.8	0.9	[2.53, 3.07]
Criminologist 1	2.7	0.8	[2.45, 2.95]
Criminologist 2	2.9	0.9	[2.65, 3.15]
Criminologist 3	2.8	0.7	[2.55, 3.05]
AI models (overall)	3.4	0.7	[3.19, 3.61]
Claude AI	3.6	0.8	[3.29, 3.91]
ChatGPT	3.3	0.7	[3.02, 3.58]
Gemini	3.2	0.6	[2.95, 3.45]

*Note:* Each assessor (human and AI) evaluated all 10 cases, generating 60 total assessments.

The data indicate that AI models consistently assigned higher risk scores compared to human assessors, with an average score of 3.4 versus 2.8, respectively. Among the AI models, Claude AI demonstrated the most conservative approach, consistently providing the highest risk scores (*M* = 3.6, SD = 0.8).

### 4.3 Distribution of Risk Scores

The distribution analysis revealed distinct patterns in risk assessment approaches between human and AI assessors (see Table 2).

**TABLE 2:** Distribution of risk scores by assessor type (*N* = 60).

Risk level	Human assessors (%)	AI models (%)
1 (Low)	15.0	7.7
2 (Low‐medium)	25.0	17.7
3 (Medium)	30.0	27.7
4 (Medium‐high)	25.0	32.3
5 (High)	5.0	14.6
Total	100.0 (30 assessments)	100.0 (30 assessments)

*Note:* Percentages represent the distribution of 30 human assessments (3 assessors × 10 cases) and 30 AI assessments (3 models × 10 cases).

AI models demonstrated a greater tendency to assign higher risk levels, with 47% of assessments falling into levels 4 or 5. In comparison, only 30.0% of human assessments were rated at these higher levels. Conversely, human assessors more frequently assigned low‐risk ratings (levels 1–2), accounting for 40.0% of their evaluations, compared to a smaller proportion among AI models. These differences suggest that AI systems tended to adopt a more precautionary or risk‐sensitive approach, whereas human evaluators leaned toward more moderate or lower‐risk classifications.

### 4.4 Inter‐Rater Reliability

Inter‐rater reliability was examined using intraclass correlation coefficients (ICC), calculated using a two‐way random effects model for absolute agreement (see Table 3).

**TABLE 3:** Intraclass correlation coefficients for risk assessments.

Assessor group	ICC	95% CI	*N*	Cases
Human assessors	0.56	[0.41, 0.69]	3	10 Each
AI models	0.79	[0.68, 0.86]	3	10 Each

The analysis revealed moderate reliability among human assessors and higher reliability among AI models, reflecting the systematic approach of AI systems versus the natural variation in human clinical judgment.

## 5 Qualitative Analysis of Key Themes

### 5.1 Consideration of Historical Factors

Analysis of assessment patterns revealed distinct differences in how historical factors were weighted and interpreted (see Table 4).

**TABLE 4:** Frequency of historical factor consideration.

Historical factor	Human Avg	AI Avg
Prior violence	4.3	7.3
Substance abuse	3.3	6.7
Mental disorders	4.0	7.3

For instance, in Case 7, a human assessor noted: “Despite a history of violence, the patient shows significant improvement in impulse control over the past 6 months.” In contrast, Claude AI stated: “The recurring history of violence indicates a high risk of violent behavior recurrence.”

### 5.2 Treatment Recommendations

Significant differences emerged in treatment approach recommendations between human and AI assessors (see Table 5).

**TABLE 5:** Types of treatment recommendations.

Recommendation	Human Avg	AI Avg
Gradual integration	4.3	2.0
Intensive treatment	2.3	4.3
Psychiatric monitor	5.3	7.3
Vocational rehab	3.3	2.0

## 6 Discussion

This study examined how advanced LLMs and human professionals differ in their approaches to offender risk assessment within the HCR‐20V3 framework. The findings suggest that the differences between these two groups extend well beyond the assigned risk scores, reflecting more profound divergences in reasoning, interpretive logic, and the underlying conceptualization of risk itself.

One of the most prominent findings was the consistent tendency of AI‐based models to assign higher risk scores than human assessors. While this difference reached statistical significance, it raises a central question: are these models inherently conservative, or do they apply assessment criteria with greater rigidity and uniformity? This pattern is consistent with the quantitative distribution presented in Table 2, where nearly half of AI‐generated assessments (47%) fell within the “Medium‐High” to “High” range, compared to only 30% among human raters. Moreover, Table 4 indicates that AI models weighted historical risk indicators almost twice as heavily as human evaluators, reinforcing the notion that their elevated scores stem from a structural overreliance on static predictors.

This pattern aligns with previous critiques of algorithmic rigidity and bias in predictive systems (Chouldechova 2017; Chouldechova and Roth 2020), suggesting that large language models may replicate the structural emphasis on historical and static factors typical of traditional actuarial approaches. Empirically, AI models placed greater weight on variables such as prior violence, substance use, and psychiatric history, whereas human clinicians focused more on recent behavioral improvements and dynamic changes in rehabilitation. This finding highlights the limited sensitivity of algorithmic systems to dynamic indicators of change and their tendency to “play it safe” by detecting risk where humans might see growth potential. This divergence likely stems from the fundamental operational differences between algorithmic pattern recognition and clinical judgment. It is plausible that the models' heightened emphasis on static factors is less a deliberate ‘risk‐averse’ strategy and more a reflection of their training paradigm. LLMs are optimized to detect strong correlations within textual data.

Historical events, such as prior violence, are typically documented as discrete, factual data points, forming robust statistical signals. Dynamic factors, conversely, such as an individual's motivation to change, are nuanced and context‐dependent. Consequently, the models may be systematically overweighting historical data, not because they “understand” it to be more important, but because it constitutes a more precise pattern within their probabilistic framework.

At the same time, AI systems exhibited higher inter‐rater reliability, likely because they are not influenced by emotional bias, fatigue, or contextual ambiguity. While this numerical consistency reflects the algorithmic uniformity of model behavior, it also highlights a limitation: reliability does not necessarily imply validity. Furthermore, this finding warrants a balanced interpretation. One perspective is that AI models are consistently reproducing overly cautious judgments.

An alternative view, however, is that human variability might reflect not only sensitivity to nuance, but also susceptibility to cognitive biases, such as an optimism bias or a recency effect, which could lead to an underestimation of risk. From this standpoint, the AI's consistency could be seen not as a flaw, but as a potential anchor against subjective drift in human judgment. While this consistency may be viewed as an advantage—reflecting stability and reproducibility—it can also become a limitation when flexibility and contextual responsiveness are required.

The consistency of AI provides a stable foundation that can anchor human judgment and reduce subjectivity; however, it must be balanced by human interpretation, which introduces context, empathy, and ethical reflection. The key challenge is to combine these strengths—algorithmic consistency and clinical flexibility without losing the capacity to recognize change, nuance, and potential for rehabilitation. The qualitative findings further reinforce this distinction. AI models relied heavily on static historical factors and often interpreted them as definitive indicators of future violence risk, whereas human assessors emphasized recent progress and rehabilitation potential.

These differences are not merely procedural; they reflect contrasting worldviews regarding human behavior and the potential for transformation. While algorithms operate within probabilistic frameworks, human professionals bring contextual understanding and an appreciation of human variability and growth. In this sense, the contrast between AI and human reasoning is as philosophical as it is methodological.

Ethical divergences also emerged. The reasoning gap mirrors concerns raised by Elyosef et al. (2024) and Rudin (2019) regarding the need for interpretable, value‐aligned models in high‐stakes decisions. In the present study, AI‐generated evaluations lacked explicit acknowledgment of contextual, social, or cultural dimensions, underscoring the continued need for human oversight—not merely for validation, but for preserving moral and clinical depth. Without this human dimension, risk assessment risks becoming a purely technical exercise, detached from the ethical foundations of forensic and clinical practice. These differences were also reflected in treatment recommendations.

AI models tended to favor more cautious and intensive interventions, such as inpatient treatment or enhanced monitoring, while human experts were more inclined toward rehabilitation and gradual community reintegration. This divergence represents two fundamentally different orientations toward risk management: one focused on control and containment, the other on empowerment and change. The implications of favoring one approach over the other extend beyond clinical outcomes to broader societal conceptions of justice, responsibility, and human agency.

The hybrid model proposed in this study—where AI first produces a standardized, data‐driven baseline assessment and the clinician refines it through contextual and ethical interpretation—builds on efforts to standardize risk communication within forensic and clinical settings (van Dooren et al. 2024). Such integration bridges the gap between algorithmic reproducibility and professional judgment, combining the precision of machine analysis with the depth and adaptability of human expertise. It represents a step toward a more coherent and ethically grounded framework for clinical risk assessment. Conceptually, this hybrid model can be viewed as an emerging framework of augmented judgment, wherein algorithmic and human reasoning function as complementary epistemic systems. Such a perspective moves beyond the dichotomy of “human versus machine” toward an integrated paradigm of shared cognitive responsibility in forensic decision‐making.

Nevertheless, it is essential to avoid glorifying technology at the expense of human insight. Although AI models are technologically impressive, they remain blind to moral and emotional nuances. Successful integration will require not only technological refinement but also the establishment of robust ethical, legal, and cultural frameworks to ensure the responsible and equitable use of these technologies. In this sense, the contribution of the present study is twofold: it highlights the promise of AI as a consistent and objective support tool, while emphasizing the enduring importance of human judgment as the ultimate interpretive and moral compass in forensic and clinical decision‐making.

## 7 Conclusions

This study makes a novel and important contribution to the evolving discourse on the role of artificial intelligence in clinical risk assessment. Through a direct comparison of advanced AI models and human professionals, it exposes consistent differences in how risk is conceptualized, structured, and acted upon. AI models demonstrated greater internal consistency and a tendency to overestimate risk, while human assessors displayed higher contextual sensitivity and responsiveness to dynamic change.

However, this should not be framed as a competition between “man and machine,” but rather as an opportunity for collaboration. A hybrid model may produce more accurate, individualized, and ethically sound risk evaluations. Such a model could be implemented in two stages: First, the AI would generate an initial, data‐driven risk report, flagging key historical factors and providing a consistent baseline score. Second, the clinician would use this report not as a final verdict, but as a decision‐support tool. The expert would then integrate their own clinical insights, consider dynamic and contextual factors the AI may have missed, and formulate the final, nuanced assessment. In this framework, AI is not the decision‐maker but a tool that enhances consistency and helps mitigate bias. Advancing this integration will require studies using real‐world clinical data, culturally adapted models, and robust ethical frameworks. The aim is not technological supremacy, but human‐centered risk assessment.

## Author Contributions

Shai Farber conceived the study, conducted the literature review, and drafted and revised the manuscript.

## Funding

The author has nothing to report.

## Conflicts of Interest

The author declares no conflicts of interest.

## Supporting information


Supporting Information S1
