# Supplementary material for: Human Experts and AI Models in Offender Risk Assessment: A Comparative Pilot Study Using the HCR‐20V3
Source: Behav Sci Law. 2025 Nov 11;44(1):87–95. doi: 10.1002/bsl.70023 (PMC12865665; doi:10.1002/bsl.70023)
Supplement: Supplementary file 1 — Supporting Information S1 [file BSL-44-87-s001.pdf]

Appendices

A. Detailed description of the HCR-20 assessment tool

The Historical-Clinical-Risk Management-20 (HCR-20V3; Douglas et al., 2013) is a comprehensive violence risk assessment tool used in forensic settings. It evaluates 20 risk factors across three domains:

Historical (H1-H10): broad historical risk-related domains commonly discussed in the HCR-20V3 literature, including histories of violence, antisocial behavior, substance use, mental disorder, personality pathology, psychosocial functioning, and prior responses to intervention.

Clinical (C1-C5): current and recent dynamic clinical factors such as insight, violent ideation, active mental health symptoms, emotional and behavioral instability, and responsiveness to treatment.

Risk Management (R1-R5): future-oriented contextual and management considerations, including professional supervision and services, living circumstances, social support, treatment adherence, and stress and coping capacity.

Each factor is rated 0 (absent), 1 (possibly present), or 2 (present). The tool combines structured assessment with professional judgment, integrating both static and dynamic risk factors.

B. Sample anonymized case report (with all identifying information removed)

Background: The subject is a 35-year-old individual with a history of mental health issues and substance abuse. They have been referred for a risk assessment following a recent altercation at a rehabilitation facility.

Relevant History:

- First diagnosed with bipolar disorder at age 22
- History of alcohol and stimulant abuse, with periods of sobriety
- Two prior arrests for disorderly conduct, no convictions
- Unstable employment history, longest job held for 18 months

Current Situation:

- Recently discharged from a 30-day inpatient program for substance abuse
- Medication compliance has been inconsistent in the past month
- Reported increased irritability and difficulty sleeping
- Altercation at the rehabilitation facility involved verbal threats, no physical violence

Support System:

- Lives alone, but has a supportive sister who checks in regularly
- Attends weekly group therapy sessions
- Has expressed interest in vocational training programs

Risk Factors:

- History of impulsivity during manic episodes
- Inconsistent medication adherence
- Ongoing struggles with substance abuse
- Limited coping skills in high-stress situations

Protective Factors:

- No history of severe violence
- Willingness to engage in treatment
- Supportive family member
- Interest in self-improvement and vocational training

C. Coding guide for content analysis

Domain	Components	Assessment Criteria
**Risk Factors**	Historical (H1-H10)	Violence, Age at First Incident, Relationships, Employment, Substance Use, Mental Illness, Psychopathy, Early Maladjustment, Personality, Supervision
	Clinical (C1-C5)	Insight, Attitudes, Current Symptoms, Impulsivity, Treatment Response
	Risk Management (R1-R5)	Plans, Destabilizers, Support, Compliance, Stress
**Protective Factors**	Individual	Treatment Response, Coping Skills
	Environmental	Social Support, Employment/Education
	Motivational	Change Readiness

Domain	Components	Assessment Criteria
**Assessment Scale**	Risk Levels	1 (Low) → 2 (Low-Medium) → 3 (Medium) → 4 (Medium-High) → 5 (High)
**Analysis Focus**	Comparative Elements	Human vs. AI Patterns, Factor Weights, Treatment Recommendations
	Special Attention	Inter-rater Gaps (≥2 points), AI-specific Patterns, Protection vs. Risk Balance
**Documentation**	Required Elements	Factor Frequency, Context Notes, Supporting

D. Technical Details on AI Models

Claude AI (Anthropic): Claude AI, developed by Anthropic, is a large language model based on an advanced transformer architecture. It is trained on a diverse corpus of texts, including books, articles, and filtered internet content. The model undergoes rigorous pre-processing steps, including content filtering and removal of personally identifiable information. Claude's training data is continuously updated, with the latest version including information up to April 2024. Known limitations include potential biases in language generation and occasional factual inconsistencies in rapidly evolving topics. For this study, Claude was used with task-specific prompt design and iterative instruction refinement based on anonymized synthetic clinical case reports, without any modification to the underlying model parameters.

Source: Anthropic. (2024). Claude AI: Technical specifications and capabilities. https://www.anthropic.com/claude-ai-specs

ChatGPT (OpenAI): ChatGPT, developed by OpenAI, utilizes the latest iteration of the GPT (Generative Pre-trained Transformer) architecture. Its training data encompasses a vast array of online text sources, including academic papers, books, and websites, updated through 2024. The model employs advanced data cleaning techniques, content moderation, and anonymization of personal data. For this study, ChatGPT was used with tailored prompt instructions designed to elicit structured professional judgment-style responses to the synthetic case vignettes, without access to proprietary assessment materials.

Source: OpenAI. (2024). ChatGPT: Model Card and Technical Documentation. https://openai.com/chatgpt-tech-docs

Gemini (Google): Google's Gemini is a multimodal AI model that can process and generate text, images, and other data types. Its training data includes a wide range of multimedia sources, continuously updated to include current information as of 2024. Gemini employs sophisticated pre-processing techniques, including content filtering, data normalization, and format standardization. For this research, only Gemini's text-based capabilities were used to generate structured analytical responses to the synthetic case materials.

Source: Google AI. (2024). Gemini: Technical overview and latest developments. https://ai.google/gemini-technical-overview

Pre-processing steps for case reports: All case reports underwent a standardized pre-processing procedure before being input into the AI models. This included thorough anonymization to remove all identifying information, standardization of format and terminology, segmentation into relevant sections (such as history, status, and risk factors), and a careful review of language to minimize emotionally charged or judgmental phrasing. These steps ensured consistency in input data across all AI models and human assessors, while maintaining the integrity and relevance of the clinical information.
